# Synthesis, Characterization, and Biological Activity of *N* ^′^-[(*Z*)-(3-Methyl-5-oxo-1-phenyl-1,5-dihydro-4*H*-pyrazol-4-ylidene)(phenyl)methyl]benzohydrazide and Its Co(II), Ni(II), and Cu(II) Complexes

**DOI:** 10.1155/2014/718175

**Published:** 2014-09-15

**Authors:** Jonnie N. Asegbeloyin, Oguejiofo T. Ujam, Emmanuel C. Okafor, Ilknur Babahan, Esin Poyrazoglu Coban, Ali Özmen, Halil Biyik

**Affiliations:** ^1^Department of Pure and Industrial Chemistry, University of Nigeria, Enugu State, Nsukka 410001, Enugu State, Nigeria; ^2^Department of Chemistry, Adnan Menderes University, 09010 Aydin, Turkey; ^3^Department of Biology, Adnan Menderes University, 09010 Aydin, Turkey

## Abstract

Reaction of 1-phenyl-3-methyl-4-benzoyl-pyrazol-5-one and benzoyl hydrazide in refluxing ethanol gave *N* ^′^-[(*Z*)-(3-methyl-5-oxo-1-phenyl-1,5-dihydro-4*H*-pyrazol-4-ylidene)(phenyl)methyl]benzohydrazide (HL^1^), which was characterized by NMR spectroscopy and single-crystal X-ray structure study. X-ray diffraction analyses of the crystals revealed a nonplanar molecule, existing in the keto-amine form, with intermolecular hydrogen bonding forming a seven-membered ring system. The reaction of HL^1^ with Co(II), Ni(II), and Cu(II) halides gave the corresponding complexes, which were characterized by elemental analysis, molar conductance, magnetic measurements, and infrared and electronic spectral studies. The compounds were screened for their *in vitro* cytotoxic activity against HL-60 human promyelocytic leukemia cells and antimicrobial activity against some bacteria and yeasts. Results showed that the compounds are potent against HL-60 cells with the IC_50_ value ≤5 *μ*M, while some of the compounds were active against few studied Gram-positive bacteria.

## 1. Introduction

Compounds derived from 4-acylpyrazol-5-one have continued to receive considerable attention due to their pharmaceutical importance and high biological activity [1–3]. They also enjoy a range of applications as a substructure in dye chemistry and synthesis [4]. The facile incorporation of conjugated systems with special features into the pyrazolone core leads to the formation of compounds with intense colour. Hydrazones and other Schiff bases are among the most studied 4-acylpyrazol-5-one derivatives, more so considering the fact that molecules with hydrazine moiety have been noted for their biological activity [5], especially as potential inhibitors for many enzymes [6]. The interest in the chemistry of 4-acylpyrazol-5-one Schiff bases stems from their interesting ligating ability due to the presence of multielectron rich donor atoms [7–9]. This is augmented by their tendency to exist in various tautomeric forms [10–12]. The ability of this class of compounds to form coordination compounds with a wide range of transition metals has been utilized in analytical chemistry, where they have been used as effective chelating and extracting reagents for many metal ions [13–15]. The literature is replete with studies of 4-acylpyrazol-5-one Schiff bases and their metal complexes. However, the study of biological activities of this class of compounds and their metal complexes is largely restricted to studies on microorganisms. Few reports are available in the literature on the study of their anticancer properties [16, 17]. With the increasing search for nonplatinum based anticancer drugs with less side effects, and similar, or better cytotoxicity, it is imperative to investigate the possibility of the use of 4-acylpyrazolone derivatives for cancer chemotherapy. In this paper, we report synthesis and characterization of* N*′-[(Z)-(3-methyl-5-oxo-1-phenyl-1,5-dihydro-4*H*-pyrazol-4-ylidene)(phenyl)methyl]benzohydrazide and its Co(II), Ni(II) and Cu(II) complexes, the* in vitro* antimicrobial activity against some bacteria and yeasts, and their antiproliferative effect on human promyelocytic leukemia cells.

## 2. Experimental

### 2.1. Chemicals and Instrumentation

All the solvents are of analytical grade and were used without further purification. 1-phenyl-3-methylpyrazol-5-one, methyl benzoate and hydrazine mono-hydrate were used as supplied by Fluka. 1-phenyl-3-methyl-4-benzoyl-pyrazol-5-one and benzoylhydrazide were synthesized in accordance with reported procedures [18, 19]. Elemental analyses of C, H and N were performed using Carlo Erba Elemental analyzer EA 1108. Melting points were obtained with a Fisher John melting point apparatus. Magnetic moments were done on a magnetic susceptibility balance—Sherwood Scientific Cambridge, Model No.MK-I. The molar conductance measurements of the metal complexes were done in DMF/DMSO using Innolab conductivity meter Level 1. The percentage of metal in the complexes was determined using an Agilent ICP-MS7500Ce. IR spectra were recorded on a Perkin Elmer Spectrum 100 at the University of Waikato, Hamilton, New Zealand. ^1^H and ^13^C NMR spectra were obtained from a Bruker AV 500 MHz for ^1^H and 125 MHz for ^13^C using a 5 mm quadra nuclei probe (QNP). The X-ray crystallographic data were obtained at the University of Auckland on a Bruker SMART APEX II diffraction equipped with a CCD area detector, using MoKα radiation (*λ* = 0.71073 Å). The software SMART was used for the collection of data frames, for indexing reflections, and to determine lattice parameters; SAINT was used for the integration of the intensity of the reflections and for scaling [20]. SADABS was used for empirical absorption correction [21]. The structures were solved using SHELXS-97, and SHELXL-97 was also used for structure refinements and reporting [22]. The structures were refined by full-matrix least-squares based on F_o_^2^ with anisotropic thermal parameters for non-hydrogen atoms.

### 2.2. Synthesis of *N*′-[(Z)-(3-Methyl-5-oxo-1-phenyl-1,5-dihydro-4*H*-pyrazol-4-ylidene)(phenyl)methyl] benzohydrazide (HL^1^)

Synthesis and crystallization of HL^1^ was done by a modification of literature procedure [23] as follows.

**Figure 1:**
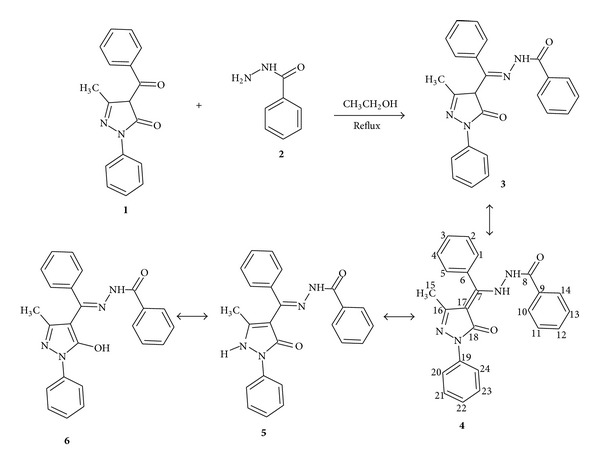
Synthesis of HL^1^.

A solution of 4-benzoyl-3-methyl-1-phenylpyrazol-5-one (2.78 g; 0.01 mol) in 30 mL ethanol was mixed with a solution of benzoyl hydrazide (1.36 g; 0.01 mol) in ethanol (20 mL). The mixture refluxed for 2 h and cooled (Figure 1). The yellow precipitate formed was isolated by gravity filtration and recrystallized from ethanol. Crystals suitable for X-ray crystallographic analysis were obtained by slow dissolution of HL^1^ in ethanol by warming and addition of few drops of dimethyl formamide (DMF), followed by slow evaporation of the solution at room temperature for 1 week.

### 2.3. Synthesis of Ni(II), Cu(II) and Co(II) Metal Complexes of HL^1^

To an ethanolic solution (20 mL) of* N*′-[(Z)-(3-methyl-5-oxo-1-phenyl-1,5-dihydro-4*H*-pyrazol-4-ylidene)(phenyl)methyl]benzohydrazide (0.43 g, 0.001 mol), ethanolic solution (10 mL) of the metal chloride (0.001 mol) was added with constant stirring. The coloured mixture was then refluxed for 4 h. The resulting metal complex was filtered hot, washed with boiling mixture of 1 : 1 water/ethanol, dried under suction, and kept in vacuum over CaCl_2_.

### 2.4. Microorganisms and Condition for Cultivation

Fifteen bacteria and five yeast cultures were used in the study. Ten bacterial strains and two yeast strains were obtained from the American Type Culture Collection (ATCC, Rockville, MD, USA). Other microorganism strains were obtained from Adnan Menderes University Faculty of Medicine. The Gram-negative (G−) were* Escherichia coli* ATCC 25922,* Salmonella typhimurium* ATCC 14028,* Proteus *sp.,* Serratia marcescens*, and* Enterobacter *sp. and the Gram-positive (G+) were* Micrococcus luteus* ATCC 9341,* Staphylococcus aureus* ATCC 25923,* Staphylococcus epidermidis* ATCC 12228,* Bacillus cereus* ATCC 11778,* Bacillus subtilis* ATCC 6633,* Bacillus thuringiensis*,* Enterococcus faecalis* ATCC 29212,* Enterococcus faecalis* ATCC 51299,* Streptococcus pneumoniae* ATCC 49617, and* Listeria monocytogenes*. Also, yeast strains such as* Candida utilis*,* Candida albicans* ATCC 90028,* Candida glabrata*,* Candida tropicalis*, and* Saccharomyces cerevisiae* ATCC 9763 were used.* Escherichia coli* ATCC 25922,* Staphylococcus aureus* ATCC 25923,* Staphylococcus epidermidis* ATCC 12228,* Salmonella typhimurium* ATCC 14028,* Listeria monocytogenes*,* Proteus *sp.,* Serratia marcescens*, and* Enterobacter *sp. were cultured in nutrient broth (NB) (Merck) at 37°C;* Streptococcus pneumoniae* ATCC 49617,* Enterococcus faecalis* ATCC 29212, and* Enterococcus faecalis* ATCC 51299 were cultured in brain heart infusion broth (BHIB) (Merck) at 37°C for 24 h.;* Micrococcus luteus* ATCC 9341,* Bacillus cereus* ATCC 11778,* Bacillus subtilis* ATCC 6633, and* Bacillus thuringiensis* were cultured in nutrient broth (NB) (Merck) at 30°C;* Candida utilis*,* Candida albicans* ATCC 90028,* Candida glabrata*,* Candida tropicalis*, and* Saccharomyces cerevisiae* ATCC 9763 were cultured in malt extract broth (MEB) (Merck, USA) at 30°C for 24 h.

### 2.5. Antimicrobial Assay

The antimicrobial activities of all the synthesized compounds were determined by the disc diffusion method [24, 25], and the minimum inhibitory concentration (MIC) was obtained by broth dilution method [25].

### 2.6. Disc Diffusion Method

The standard method of Antimicrobial Disc Susceptibility Tests reported by the National Committee for Clinical Laboratory Standards [25] was used. Fresh stock solutions (1000 *μ*g*·*mL^−1^) of all the synthesized compounds were prepared in DMSO. The inoculum suspensions of the tested bacteria and yeasts were prepared from the broth cultures (18–24 h) and the turbidity equivalent adjusted to 0.5 McFarland standard tube to give a concentration of 1 × 10^8^ bacterial cells and 1 × 10^6^ yeast cells/mL. To test the antimicrobial activity of all the synthesized compounds, a Mueller Hinton Agar (MHA) plate was inoculated with 0.1 mL broth culture of bacteria or yeast. Then a hole of 6 mm in diameter and depth was made on top with a sterile stick and filled with 50 *μ*L of synthesized compounds. Plates inoculated with* E. coli* ATCC 25922,* S. typhimurium* ATCC 14028,* S. aureus* ATCC 25923,* S. epidermidis* ATCC 12228,* E. faecalis* ATCC 29212,* Enterococcus faecalis* ATCC 51299,* S. pneumoniae* ATCC 49617,* Listeria monocytogenes*,* Proteus *sp.,* S. marcescens*, and* Enterobacter *sp. were incubated at 37°C for 24 h, while those with* M. luteus* ATCC 9341,* Bacillus subtilis* ATCC 6633,* B. cereus* ATCC 11778,* B. thuringiensis*,* S. cerevisiae* ATCC 9763,* C. albicans* ATCC 90028,* C. glabrata*,* C. utilis*, and* C. tropicalis* were incubated at 30°C for 24 h. At the end of incubation time, the diameters of the inhibition zones formed on the MHA were evaluated in millimeters. Disc of chloramphenicol (C30), gentamicin (CN10), tetracycline (TE30), erythromycin (E15), ampicillin (AMP10), and nystatin (NS100) were used as positive controls. The measured inhibition zones of the study compounds were compared with those of the reference discs.

### 2.7. Minimum Inhibitory Concentration (MIC)

Minimum inhibitory concentration (MIC) was determined by reported method [25]. The study bacteria were inoculated in nutrient broth and incubated at 30–37°C for 24 hr while the yeasts were inoculated in Malt Extract Broth and incubated at 30°C for 48 h. The inoculums were adjusted according to 0.5 McFarland standard tube. Initially, 100 *μ*L of Mueller Hinton Broth (MHB) was placed in each well. After, the compounds were dissolved in DMSO (2 mg mL^−1^) and transferred into the first well. Two fold serial dilution of the compounds were carried out to determine the MIC, within the concentration range 256 to 0.125 *μ*g mL^−1^. Cultures were grown at 30–37°C (18–20 h) and the final inoculum was approximately 10^6^ cfu mL^−1^. The lowest concentration of the study compounds that resulted in complete inhibition of the microorganisms was represented as MIC (*μ*g mL^−1^). Similar procedures were done on positive controls, streptomycin (I. E. Ulagay). In each case, the test was performed in triplicate and the results were expressed as means.

### 2.8. Cell Antiproliferation Test

HL-60 promyelocytic leukaemia cells were purchased from ATCC. Cells were grown in RPMI-1640 medium supplemented with 10% heat inactivated fetal calf serum, 1% L-glutamine, and 1% penicillin/CO_2_. All media and supplements were obtained from Life Technologies. HL 60 cells were seeded in T-25 tissue culture flasks at a concentration of 1 × 10^5^/mL and incubated with increasing concentrations (5, 10, 20, and 40 *μ*M) of the study compounds. Cell counts and IC_50_ values were determined at 24 and 72 h using a Thoma slide. Experiments were done in triplicate. The percentage of cell divisions compared to the untreated control was calculated as follows:

(1) [(C72 h+drug−C24 h+drug)(C72 h−drug−C24 h−drug)]×100 =%  cell  division,

where C72 h + study compound is the cell number after 72 h of treatment with study compound, C24 h + compound is the cell number after 23 h of treatment with study compound, C72 h − compound is the cell number after 72 h without treatment with compound, and C24 h − compound is the cell number after 24 h without treatment with compound.

## 3. Results and Discussion

### 3.1. X-Ray Crystal Structure of HL^1^

**Figure 2:**
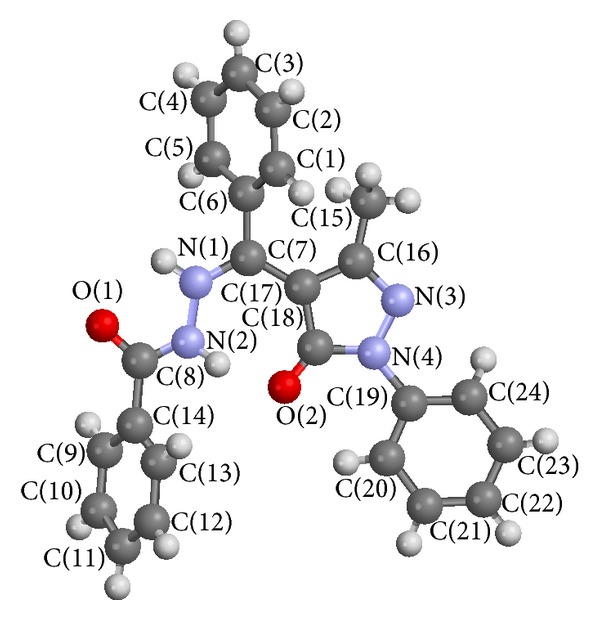
Molecular structure of HL^1^ showing the atom numbering scheme with thermal ellipsoids at the 50% probability level.

**Figure 3:**
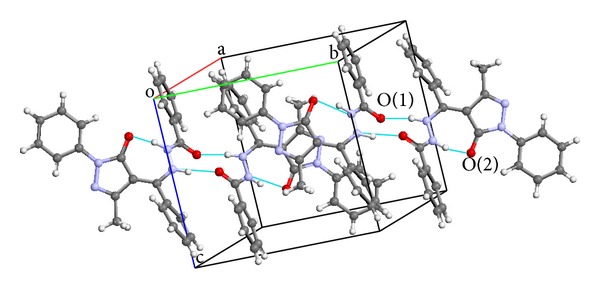
A view of the crystal packing of HL^1^ showing the intramolecular and intermolecular hydrogen bonding.

The crystal structure of HL^1^ has been reported in literature [23] but was redetermined to investigate if the new method of crystal isolation could affect the R-factor which was earlier reported as 0.0542 [23], and control the intramolecular rearrangement of the molecule from structure (4) to any of the other tautomers, see Figure 1. The data and structure refinement parameter details are given in Table 2. The crystal structure of HL^1^ was solved by direct method. The nitrogen and oxygen atoms were located followed by other atoms in subsequent refinements. All nonhydrogen atoms were refined as anisotropic. The X-ray crystal structures and atom numbering schemes of HL^1^ are as shown in Figure 2. The selected bond lengths and angles are shown in Table 1. The crystal data and structure refinement details are presented in Table 2. The compound is a nonplanar molecule. The measured angle between the pyrazolone ring plane C(18) C(17) C(16) N(3) and C(6) C(1) C(5) plane is 71.4°. In addition the angle between C(8) N(2) N(1) and C(6) C(7) N(1) planes is 33.6°. The phenacyl group adopts a gauche conformation about the N—N vector. The angle between the phenacyl C(13) C(14) C(9) and N(2) C(14) C(8) planes is 29.9°. The hydrazino nitrogen atoms adopted the trans-conformation which is more preferred when not in a ring system [26, 27]. The bond length of hydrazino (>N–N<) nitrogen atoms 1.3820(9) Å the carbonyl (>C=O) group 1.2396(9) Å is in agreement with literature reports [23, 28]. The ring carbon–oxygen O(2)–C(18)bond length 1.2655(9) Å is longer but in good agreement with earlier reports on the pyrazolone ring carbonyl group [23, 29]. The molecular structure of the compound indicated intermolecular hydrogen bonding between the hydrogen atom on N(2) and the O(2) forming a non planar seven membered ring system. Intramolecular hydrogen bonding is also observed in the molecule as shown in the crystal packing motif in Figure 3.

### 3.2. Analytical and Physical Data

All the metal complexes are colored and soluble in polar solvents (dimethyl sulfoxide, dimethyl formamide, and anhydrous ethanol) but insoluble in solvents such as hexane, pentane, tetrahydrofuran and carbon tetrachloride. Analytical and physical data of all the study compounds are shown in Table 3. The complexes are nonelectrolytes in DMSO, as evident from the molar conductivity values which range from ~9 to 13 Ohm^−1^ cm^2^ mol^−1^ [30–32].

### 3.3. FT-Infrared Spectra

The relevant stretching frequencies are tabulated in Table 4. The absorption bands in the region ~3000–3280 cm^−1^ in the spectral of HL^1^ and the metal complexes were assigned to NH stretching vibrations. Two strong* v*(C=O) bands are observable in the keto-amine form of HL^1^, one assignable to* v*(C=O) of the lateral chain comprising the hydrazide moiety and the other to the* v*(C=O) of pyrazolone ring [33–35]. These strong vibrational bands (cm^−1^) were observed at 1645 and 1596, respectively. The* v*(C=O) band assigned to the hydrazide moiety shifted to lower frequencies in the metal complexes, suggesting that the carbonyl oxygen was involved in coordination to the metal ions [36–38]. Bands in the region of 1566–1570 cm^−1^ have been assigned to imino* v*(C=N) in all the metal complexes. The bands in the region of 1437–1440 cm^−1^ in the metal complexes have been assigned to* v*(C–O) [33, 39] resulting from enolization and deprotonation before coordination [14, 40].

The bands in the region ~1025–1120 cm^−1^ in the ligand and the metal complexes have been assigned to* v*(N–N). Bands in the range ~510–520 cm^−1^ in the metal complexes were assigned to* v*(M–O) while bands in the range ~420–480 cm^−1^ were assigned to* v*(M–N) [41–43].* v*O–H of water of crystallization was observed in the region ~3370–3470 cm^−1^ in the metal complexes. HL^1^ reacted as the enol-imino form (Figure 1 (**5**)), when coordinated to metal ions in solution, coordinating via pyrazolone ring enolic (C–O), azomethine (C=N), and hydrazide side chain (C=O).

### 3.4. ^1^H and ^13^C NMR Spectra

The possible keto forms of HL^1^ are as shown in Figure 1. The X-ray structure confirmed that it crystallized in the keto-amine form (**4**). The conjugated system that extended from the pyrazolone ring confers extra stability on form (**4**). In the ^1^H NMR spectrum the 3-methyl protons signal was observed as singlet upfield at 2.17 ppm. The phenyl protons appeared as multiplets in the range 7.26–8.07 ppm while the signal due to –NH was observed at 8.83 ppm. The ^13^C-NMR spectrum of the ligand exhibited 17 carbon signals comprising 16* sp* ^*2*^ and one* sp* ^*3*^ (–CH_3_) carbons; the signal at *δ*16.06 has been assigned to carbon of the pyrazolone core side 3-methyl. Carbons of benzene rings are present at *δ* 118.47–165.18. The most deshielded carbons have been assigned relevant signals downfield. The assignments are as follows: C-1 = 127.55, C-2 = 127.42, C-3 = 129.70, C-4 = 127.42, C-5 = 127.55, C-6 = 131.43, C-7 = 138.75, C-8 = 163.64, C-9 = 130.69, C-10 = 148.27, C-11 = 132.78, C-12 = 130.04, C-13 = 128.83, C-14 = 130.04, C-15 = 15.44, C-16 = 149.28, C-17 = 120.34, C-18 = 154.55, C-19 = 138.22, C-20 = 119.85, C-21 = 129.70, C-22 = 125.26, C-23 = 129.70 and C-24 = 119.85 ppm (see (**4**) in Figure 1). All assignments were consistent with literature on related studies [44–48].

### 3.5. Electronic Spectra and Magnetic Studies

The significant electronic absorption bands in the spectra of the ligand and the metal complexes recorded in DMSO solution are presented in Table 5. The ligand shows high frequency bands at 42,533, 32,786, and 29,411 cm^−1^ which were assigned to n → π* and *π* → π* transitions [49, 50]. For a typical d^9^ octahedral Cu^2+^ complex the ^2^D free-ion term is split into two levels by the O_h_ symmetry and further split on distortion to D_4h_ or C_4v_ symmetry [51]. Three spin allowed transitions can be observed in the visible and near IR regions. In the present study the Cu(II) complex show bands at 16,354, 13,424 and 22,722 cm^−1^ which have been assigned to ^2^B_1g_→^2^B_2g_,^2^B_1g_→^2^A_1g_ and ^2^B_1g_→^2^E_g_ transitions respectively [52–54]. The observed magnetic moment of 1.90 B.M is suggestive of an octahedral geometry for Cu(II) complex [54]. Ni(II) with d^8^ configuration in an octahedral field has a ground state of ^3^F, which is split into ^3^A_2g_, ^3^T_2g_, and ^3^T_1g_(F) states and the excited state designated as ^3^T_1g_(P). Three bands can be observed in the spectra. The Ni(II) complex displays three bands at 12,545, 14,174, and 23,809, cm^−1^ assignable to ^3^A_2g_→^3^T_2g_(F), ^3^A_2g_→^3^T_1g_(F), and ^3^A_2g_→^3^T_1g_(P) transitions, respectively [55]. The magnetic moment of 2.93 B.M for Ni(II) complex is an indication of its octahedral geometry. The three bands observed at 21,276, 20,408, and 10,204 cm^−1^ for Co(II) complexes have been assigned to ^4^T_1g_(F)→^4^T_1g_(P), ^4^T_1g_(F)→^4^A_2g_(F) and ^4^T_1g_(F)→^4^T_2g_(F) transitions, respectively [52]. The magnetic moment of 4.94 B.M and the observed electronic transitions indicate high spin octahedral geometry of the complex [56].

**Figure 4:**
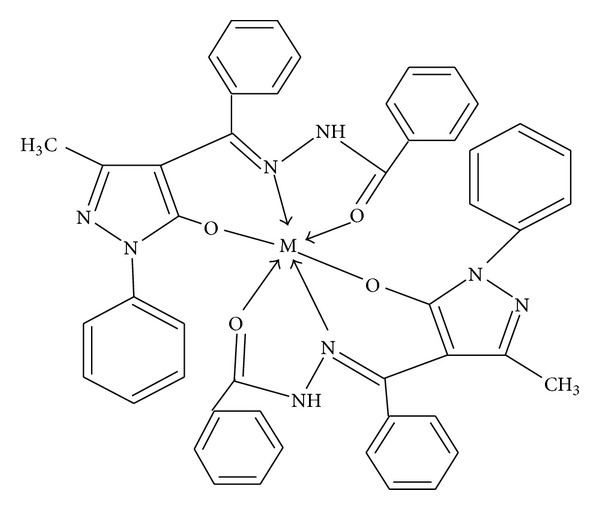
Proposed structures of the metal complexes {M=Co(II), Cu(II), Ni(II)}.

On the basis of microanalytical data, magnetic moments, conductivity measurements, and spectral analysis, Figure 4 has been proposed for the metal complexes.

### 3.6. Antimicrobial Test Results

The results of antimicrobial activities of the study compounds reported as inhibition zone diameter (mm) are showed in Table 6. The inhibition zone diameter of the reference antibiotics used on the test microorganisims are presented in Table A which can be found in Supplementary Material available online at http://dx.doi.org/10.1155/2014/718175. The MIC values which suggested that some of the studied compounds exhibited appreciable antimicrobial activity are showed in Table 7. The ligand, Co(II) and Cu(II) complexes showed appreciable activities against some pathogen bacteria, while the Ni(II) complex did not show any antimicrobial activity. The ligand (HL^1^) showed significant antimicrobial activity against four Gram- positive bacteria* Micrococcus luteus* ATCC 9341,* Staphylococcus aureus* ATCC 25923,* Bacillus cereus* ATCC 11778, and* Bacillus subtilis* ATCC 6633; the Co complex showed antimicrobial activity against two Gram-positive bacteria* Bacillus cereus* ATCC 11778 and* Bacillus subtilis* ATCC 6633. Cu(II) complex exhibited antimicrobial activity against three Gram-positive bacteria* Micrococcus luteus* ATCC 9341,* Staphylococcus aureus* ATCC 25923, and* Bacillus cereus* ATCC 11778. However, none of the study compounds exhibited antimicrobial activity against tested Gram-negative bacteria and yeasts. Gram-negative bacteria are noted for the protective lipopolysaccharide (LPS) in the outer membrane of their cell walls, which protects the sensitive inner membrane and the cell wall from drugs and dyes [57]. This protective lipopolysaccharide is absent in Gram-positive bacteria. In order for these compounds to exert bacteriostatic or bactericidal actions, they must access intracellular targets [58, 59]. Therefore in Gram-negative bacteria, these compounds must cross the outer membrane, a substantial permeability barrier and thus a major determinant of antimicrobial resistance in these bacteria [59]. The observed results of the antimicrobial tests suggest that the compounds were not able to permeate the protective outer membrane of the Gram negative bacteria to the extent of having antimicrobial effects. The observed better antimicrobial action exhibited by the ligand may be ascribed to the formation of hydrogen bonds with the active centre of cell constituents, resulting in interference with the normal function of the cell [60]. On coordination, the site for hydrogen bonding is minimized in the resultant metal complexes.

MICs were measured for the compounds that showed appreciable growth inhibition zones; the study was restricted to microorganisms that were affected by the compounds. From the results of MIC tests shown in Table 7, it can be seen that MIC of selected compounds against bacterial pathogens varied from 4 to 128 *μ*g mL^−1^. The lowest MICs were observed for HL^1^ (4–8 *μ*g mL^−1^) against the four tested microorganisms. Low MICs were also recorded for Cu complex (*Staphylococcus aureus* ATCC 25923) and Co complex (*Bacillus cereus* ATCC 11778). The results suggest that the compounds showed good antimicrobial activity against these organisms.

### 3.7. Results of Cell Proliferation Test

**Figure 5:**
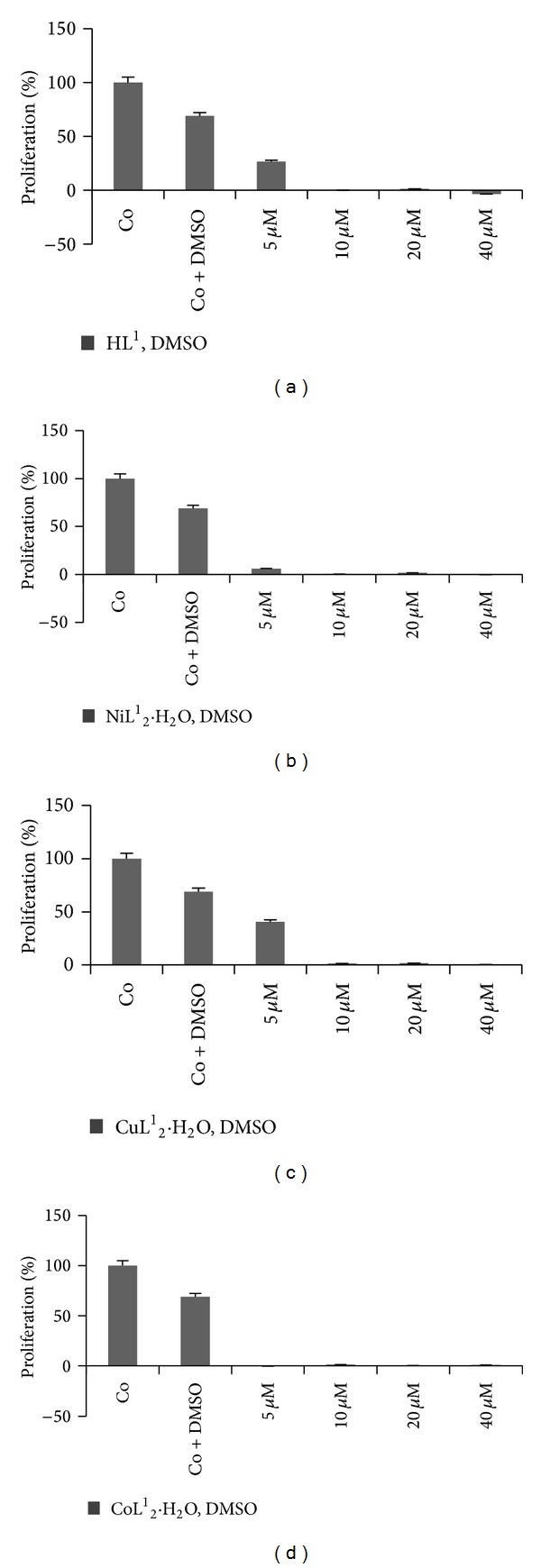
Inhibitory percentage (%) of compounds at 5–40 *μ*M on HL-60 cancer cell line.

HL-60 (*human promyelocytic leukemia cells*) cell line is a leukemic cell line that has been used for laboratory research. The HL-60 cultured cell line provides a continuous source of human cells for studying the molecular events of myeloid differentiation and the effects of physiologic, pharmacologic, and virologic processes. The results of antiproliferative activities of investigated compounds against human cancer (HL-60) cell line are presented in Table 8. The results indicated that HL^1^ and its Cu(II) complex exhibit cytotoxic effects at the lowest tested concentration with IC_50_ value between ~3 and 5 *μ*M while the Co(II) and Ni(II) complexes exhibit better cytotoxic effects with IC_50_ value ~2-3 *μ*M.  Figure 5 shows effects of different concentrations of tested compounds on HL-60 cell line. It is evident from Figure 5 that all the compounds (at <5–40 *μ*M) were able to inhibit the proliferation of more than 50% of the HL-60 cell. The tested compounds can be described as potent anticancer agents due to their considerably high antiproliferative effects. This is consistent with documented facts by the US NCI screening program, that if the IC_50_ value (following the incubation of a compound and cell lines between 48 and 72 h) is less than 10 *μ*M, the compound is considered to have* in vitro* cytotoxic activity [61].

## 4. Conclusions

A 4-acylpyrazolone hydrazone and some of its metal complexes have been synthesized and characterized. The crystal and spectra data showed that the ligand crystallized in the keto-amine form and coordinated as a monoanionic tridentate ONO donor ligand. Physicochemical and spectral data show that the metal to ligand ratio is 1 : 2 in the Ni(II), Cu(II), and Co(II) complexes. The electronic data and magnetic moments are in favour of octahedral geometry for Ni(II), Cu(II), and Co(II) complexes. The compounds exhibited better antimicrobial activity against Gram-positive bacterial strains studied. The cytotoxicity test results showed that the compounds are potential anticancer agents.

## Supplementary Material

A CIF file containing complete information on the structure of HL^1^ has been deposited with CCDC, deposition number 931219, and is freely available from http://www.ccdc.cam.ac.uk/data_request/cif.

## Figures and Tables

**Table 1:** Selected bond lengths (Å) and bond angles (°) for HL^1^ (estimated standard deviations are in brackets).

Bond lengths and atomic distances (Å)			
O(1)-C(8)	1.2396(9)	N(3)-C(16)	1.3097(11)
O(2)-C(18)	1.2655(9)	N(3)-N(4)	1.4003(10)
N(1)-C(7)	1.3299(10)	N(4)-C(18)	1.3766(10)
N(1)-N(2)	1.3820(9)	N(4)-C(19)	1.4140(10)
N(2)-C(8)	1.3487(10)	C(1)-C(2)	1.3899(13)

Bond angles (°)			
C(7)-N(1)-N(2)	123.33(7)	N(1)-C(7)-C(17)	125.71(7)
C(8)-N(2)-N(1)	119.20(7)	N(1)-C(7)-C(6)	112.99(7)
C(16)-N(3)-N(4)	106.36(6)	O(1)-C(8)-N(2)	122.97(7)
C(18)-N(4)-N(3)	112.14(6)	O(1)-C(8)-C(14)	122.98(7)
C(18)-N(4)-C(19)	129.16(7)	N(2)-C(8)-C(14)	114.05(7)
N(3)-N(4)-C(19)	118.67(6)	O(2)-C(18)-N(4)	124.30(7)

**Table 2:** Crystal data and structure refinement details for HL^1^.

Identification code	HL^1^
Formula	C_24_H_20_N_4_O_2_
Formula weight	396.44
Temperature (K)	109(2)
Wavelength (Å)	0.71073
Crystal system	Triclinic
Space group	P-1
*a*/Å	9.0895(2)
*b*/Å	10.9699(3)
*c*/Å	11.0637(3)
α/°	89.5410(10)
*β*/°	78.6940(10)
*γ*/°	66.0660(10)
Volume (Å^3^)	985.59(4)
*Z*	2
Calculated density (Mg/m^3^)	1.336
Absorption coefficient (mm^−1^)	0.088
*F*(000)	416
Crystal size (mm^3^)	0.70 × 0.49 × 0.28 mm
Theta range for data collection	2.50 to 34.28°
Limiting indices	−14 ≤ *h* ≤ 13, −17 ≤ *k* ≤ 16, −16 ≤ *l* ≤ 17
Reflections collected/unique	29726/7289 [*R*(int) = 0.0274]
Completeness to theta	27.50° 100.0%
Absorption correction	Multiscan
Max. and min. transmission	0.9759 and 0.9412
Refinement method	Full-matrix least-squares on *F* ^2^
Data/restraints/parameters	7289/0/278
Goodness of fit on *F* ^2^	1.028
Final *R* indices [*I* > 2*σ*(*I*)]	*R*_1_ = 0.0439, *wR*_2_ = 0.1199
*R* indices (all data)	*R*_1_ = 0.0535, *wR*_2_ = 0.1282
Largest diff. peak and hole	0.440 and −0.289 e A^−3^

**Table 3:** Elemental analysis and physical data of the ligand and complexes.

Compound	Colour	MF	Yield %	Elemental analysis % found (calculated)				*μ*_eff_ B.M	*λ*_*M*_ ohm^−1^cm^2^mol^−1^	Melting Point °C
				C	H	N	M			
HL^1^	Yellow	C_24_H_20_N_4_O_2_	85	72.68 (72.73)	5.21 (5.05)	14.28 (14.14)	—	—	—	196
CoL^1^_2_ *·*H_2_O	Yellow	C_48_H_40_N_8_O_5_Co	70	66.34 (66.43)	4.98 (4.65)	12.99 (12.92)	6.35 (6.79)	4.93	11.56	286
NiL^1^_2_ *·*H_2_O	Green	C_48_H_40_N_8_O_5_Ni	67	66.78 (66.45)	4.81 (4.65)	13.02 (12.92)	6.60 (6.76)	2.94	12.50	272
CuL^1^_2_ *·*H_2_O	Green	C_46_H_40_N_8_O_5_Cu	60	65.61 (66.08)	5.15 (4.62)	13.41 (12.85)	7.63 (7.33)	1.90	10.34	260

**Table 4:** Relevant IR absorption bands for the ligand and metal complexes.

Compound	*v*O–H	*v*N–H	*v*C=O^a^	*v*C=O^b^	*v*C=N	*v*N–N	*v*C–O	*v*M–O	*v*M–N
L^1^	—	3132	1645	1596	—	1117	—	—	—
Cu(L^1^)_2_ *·*H_2_O	3448	3311	1597	—	1567	1026	1440	519	418
Ni(L^1^)_2_ *·*H_2_O	3376	3059	1621	—	1570	1027	1437	509	460
Co(L^1^)_2_ *·*H_2_O	3468	3275	1623	—	1566	1025	1438	510	472

^a^Hydrazide moiety, ^b^pyrazolone ring.

**Table 5:** Electronic spectra data of ligand and metal complexes.

Compounds	Bands (cm^−1^) (*ε*, Lmol^−1^cm^−1^)	Assigned transition
HL^1^	42,533 (10600)	*n*⟶π*
	32,786 (7500)	*n*⟶π*
	29,411 (9100)	*π*⟶π*

CoL^1^_2_ *·*H_2_O	21,276 (228)	^ 4^T_1g_(F) *⟶* ^4^T_1g_(P)
	20,408 (224)	^ 4^T_1g_(F) *⟶* ^4^A_2g_(F)
	10,204 (165)	^ 4^T_1g_(F) *⟶* ^4^T_2g_(F)

CuL^1^_2_ *·*H_2_O	13,424(255)	^ 2^B_1g_ *⟶* ^2^A_1g_,
	16,354(135)	^ 2^B_1g_ *⟶* ^2^B_2g_
	22,722 (245)	^ 2^B_1g_ *⟶* ^2^E_g_

NiL^1^_2_ *·*H_2_O	23,809 (225)	^ 3^A_2g_ *⟶* ^3^T_1g_(P)
	14,174 (172)	^ 3^A_2g_ *⟶* ^3^T_1g_(F)
	12,545 (142)	^ 3^A_2g_ *⟶* ^3^T_2g_(F)

**Table 6:** Antimicrobial activities of HL^1^ and the metal complexes (inhibition zone diameter (mm).

Test microorganism	HL^1^	CoL^1^_2_ *·*H_2_O	NiL^1^_2_ *·*H_2_O	CuL^1^_2_ *·*H_2_O
*Escherichia coli* ATCC 25922	—	—	—	—
*Salmonella typhimurium* ATCC 14028	—	—	—	—
*Micrococcus* *luteus, *ATCC 9341	15	—	—	12
*Staphylococcus aureus* ATCC 25923	15	—	—	14
*Staphylococcus epidermidis* ATCC 12228	—	—	—	—
*Bacillus cereus* ATCC 11778	15	13	—	10
*Bacillus subtilis* ATCC 6633	13	8	—	—
*Bacillus thuringiensis* ∗	—	—	—	—
*Enterococcus faecalis* ATCC 29212	—	—	—	—
*Enterococcus faecalis* ATCC 51299	—	—	—	—
*Streptococcus pneumoniae* ATCC 49617	—	—	—	—
*Proteus *sp.∗	—	—	—	—
*Serratia marcescens* ∗	—	—	—	—
*Enterobacter *sp.∗	—	—	—	—
*Listeria monocytogenes* ∗∗	—	—	—	—
*Candida albicans* ATCC 90028	—	—	—	—
*Candida utilis* ∗	—	—	—	—
*Candida tropicalis* ∗	—	—	—	—
*Candida glabrata* ∗	—	—	—	—
*Saccharomyces cerevisiae* ATCC 9763	—	—	—	—

(—): zone of inhibition not included in the diameter of the well.

*Special gift from Faculty of Medicine, Adnan Menderes University. **Food isolated.

**Table 7:** Minimum inhibitory concentration of compounds (MIC, *μ*g*·*mL^−1^).

Test microorganisms	HL^1^	CoL^1^_2_ *·*H_2_O	CuL^1^_2_ *·*H_2_O	Str
*Micrococcus luteus*, ATCC 9341	4	NT	16	32
*Staphylococcus aureus* ATCC 25923	4	NT	4	32
*Bacillus cereus* ATCC 11778	4	4	64	64
*Bacillus subtilis* ATCC 6633	8	128	NT	64

Note: Str: streptomycin.

(NT): Not tested.

**Table 8:** Antiproliferative activities of investigated compounds against human cancer (HL-60) cell line.

Compounds	HL^1^	CoL^1^_2_ *·*H_2_O	NiL^1^_2_ *·*H_2_O	CuL^1^_2_ *·*H_2_O
IC_50_ (*μ*M)	3.36 ± 1.12	2.52 ± 0.22	2.66 ± 0.54	4.20 ± 1.44
