# Supplementary material for: Synthesis, Characterization, and Biological Activity of N ′-[(Z)-(3-Methyl-5-oxo-1-phenyl-1,5-dihydro-4H-pyrazol-4-ylidene)(phenyl)methyl]benzohydrazide and Its Co(II), Ni(II), and Cu(II) Complexes
Source: Bioinorg Chem Appl. 2014 Sep 15;2014:718175. doi: 10.1155/2014/718175 (PMC4181943; doi:10.1155/2014/718175)
Supplement: Supplementary file 1 — A CIF file containing complete information on the structure of HL1 has been deposited with CCDC, deposition number 931219, and is freely available from http://www.ccdc.cam.ac.uk/data_request/cif. [file 718175.f1.docx]

**Supplementary material**

A CIF file containing complete information on the structure of HL^1^ has been deposited with CCDC, deposition number 931219, and is freely available from [www.ccdc.cam.ac.uk/data_request/cif](http://www.ccdc.cam.ac.uk/data_request/cif).

**Table A:** Inhibition zone diameter(mm) of the reference antibiotics to test microorganisms

| Test Microorganisms | C30 | CN10 | TE30 | E15 | AMP10 |
| --- | --- | --- | --- | --- | --- |
|  |  |  |  |  |  |
| *Escherichia coli*  ATCC 25922 | 24 | 21 | 15 | 11 | - |
| *Salmonella typhimirium*  ATCC 14028 | 17 | 16 | 15 | 8 | 8 |
| *Micrococcus luteus,*  ATCC 9341 | 25 | 15 | 26 | 30 | 28 |
| *Stapylococcus aureus* ATCC 25923 | 23 | 20 | 22 | 23 | 20 |
| *Stapylococcus*  *epidermidis*  ATCC 12228 | 22 | 17 | 19 | 11 | 17 |
| *Bacilllus cereus*  ATCC 11778 | 23 | 24 | 25 | 26 | - |
| *Bacillus subtilis*  ATCC 6633 | 22 | 20 | 12 | 25 | - |
| *Bacillus thrungiensis** | 26 | 21 | 15 | 28 | - |
| *Entereococcus*  *faecalis*  ATCC 29212 | 16 | 11 | 19 | - | 14 |
| *Entereococcus*  *faecalis*  ATCC 51299 | 15 | 12 | 24 | - | 13 |
| *Streptococcus pneumoniae*  ATCC 49617 | 24 | 20 | 15 | - | - |
| *Proteus sp.** | 17 | 24 | 16 | 20 | - |
| *Serratia marcescens** | 23 | 19 | 10 | - | 15 |
| *Enterobacter sp*.*** | 19 | 20 | 14 | - | - |
| *Listeria monocytogenes*** | 16 | 11 | 10 | - | - |

C30: Chloramphenicol (30 mg Oxoid), CN10: Gentamycin (10 mg Oxoid), TE 30:Tetracycline (30 mg Oxoid), E15: Erytromycin (15 mg Oxoid), AM10: Ampicillin (10 mg Oxoid), NS: Nystatin (100 mg Oxoid)

(-):No zone

*Special gift from Faculty of Medicine, Adnan Menderes University. **Food isolated
